# Causeway: a pipeline for genome-wide effector gene screening with Mendelian Randomization and colocalization

**DOI:** 10.1093/bioadv/vbaf110

**Published:** 2025-05-29

**Authors:** Julia A de Amorim, João Vitor F Cavalcante, Diego Marques-Coelho, Rodrigo J S Dalmolin, Vasiliki Lagou

**Affiliations:** Bioinformatics Multidisciplinary Environment, Federal University of Rio Grande do Norte, Natal, Rio Grande do Norte, 59076-550, Brazil; Bioinformatics Multidisciplinary Environment, Federal University of Rio Grande do Norte, Natal, Rio Grande do Norte, 59076-550, Brazil; Bioinformatics Multidisciplinary Environment, Federal University of Rio Grande do Norte, Natal, Rio Grande do Norte, 59076-550, Brazil; Bioinformatics Multidisciplinary Environment, Federal University of Rio Grande do Norte, Natal, Rio Grande do Norte, 59076-550, Brazil; Bioinformatics Multidisciplinary Environment, Federal University of Rio Grande do Norte, Natal, Rio Grande do Norte, 59076-550, Brazil

## Abstract

**Summary:**

The integration of quantitative trait loci and disease genome-wide association studies for pinpointing candidate causal genes is a computationally demanding task accompanied by pitfalls related to the methods used. To address these issues, we introduce Causeway, a novel Nextflow pipeline for performing summary statistics-based two sample Mendelian Randomization for causal gene prioritization. The pipeline executes sensitivity and colocalization analyses for interrogation of findings providing robust results. The tool is designed to run tasks in a computationally efficient way even in low-resource environments, such as a personal computer. Furthermore, it can scale to web servers and high-performance computing clusters.

**Availability and implementation:**

The source code of Causeway is available at GitHub https://github.com/juliaapolonio/Causeway, while the documentation and instructions to run the vignette at https://juliaapolonio.github.io/Causeway/.

## 1 Introduction

Genome-wide association studies (GWASs) have been successfully applied to discover the genetic architecture of complex and highly polygenic phenotypes (Hindorff *et al.* 2009, Uffelmann *et al.* 2021). Although GWASs have uncovered thousands of variants associated with diseases, the majority of GWAS signals are non-coding with small effect sizes overlapping regulatory elements that influence the expression of proximal or distal genes (Gaulton *et al.* 2023). Defining the regulatory function of these variants and pinpointing the effector genes at the identified loci remains a challenging task. Genetic variants affecting mRNA levels (expression quantitative trait loci, eQTLs) and protein abundance (protein quantitative trait loci, pQTLs) are among the commonly used datasets for causal gene prioritization. To date, several studies have examined the association of variants that exert a regulatory effect on gene expression in cis (cis-eQTLs) in several tissues and cell types, with summary statistics being available for downstream analyses. Similarly, publicly available pQTL data have been very informative for translational purposes, as proteins are the targets of most drugs. The integration of QTL and GWAS data using Mendelian Randomization (MR) and colocalization can enable the elucidation of putative target genes and proteins linked to GWAS signals.

MR is an approach for assessing causality between an exposure and an outcome free of confounding non-genetic factors using genetic variants as instrumental variables (IVs). When the exposure is gene expression or protein abundance, MR studies usually focus on a small genomic region using local and correlated cis-single-nucleotide polymorphisms (SNPs) as IVs (cis-MR). The validity of findings from MR is based on three assumptions: (i) the IVs must be associated with the outcome; (ii) there are no unmeasured confounders of the exposure–outcome relationship, and (iii) the IVs affect the outcome only through their effects on the exposure and not through other pleiotropic pathways. The presence of horizontal pleiotropy (i.e. IVs affect the outcome through another trait or pathway) violates the third IV assumption. One MR method proposed in the likely presence of pleiotropic effects on both exposure and outcome is the Generalized Summary-data-based MR (GSMR).

GSMR performs multi-SNP MR based on GWAS summary statistics and HEIDI (heterogeneity in dependent instruments), which removes IVs with strong putative pleiotropic effects accounting for possible linkage disequilibrium (LD) between IVs (Zhu *et al.* 2018). MR associations can be further validated by sensitivity methods, implemented in the widely used TwoSampleMR and Coloc R packages, such as tests of reverse causality (MR Steiger filtering) and heterogeneity tests, as well as co-localization to determine whether the associations with both gene expression/protein abundance and phenotype are due to the same causal variant.

The integration of molecular QTL and GWAS data at a genome-wide level using the above tools presents several challenges. Although R is an accessible and practical language, it cannot handle large-scale data. One way to mitigate this limitation is by using Rcpp to implement performance critical functions, as seen in TwoSampleMR. In contrast, GSMR, which is fully implemented in C++, offers superior computational efficiency but can present challenges with RAM management and lacks robust sensitivity analysis features. Other key challenges relate to the time-consuming installation, management of dependencies of each software/tool, and manual handling of a large number of tasks. Furthermore, the reproducibility of results can be affected by changes in software versions and system requirements across platforms (Mangul *et al.* 2019). Given these limitations, more reproducible and user-friendly tools that can accommodate various computational environments, from low-resource settings to high-performance computing clusters are necessary.

Here, we present Causeway, a novel workflow designed to flexibly and efficiently streamline genome-wide summary statistics-based two sample GSMR between any modifiable risk factor and phenotype. GSMR findings are interrogated by a range of sensitivity methods including co-localization offering a more robust approach compared to existing platforms, such as SMR portal (add reference). One of the key features of this pipeline is its ability to split the analysis of individual genes into different tasks, allowing it to run in environments with limited RAM, while still being scalable to high-performance computing systems. Scalability is achieved through Nextflow (Di Tommaso *et al.* 2017), which parallelizes tasks efficiently, reducing the processing time. This pipeline offers a complete, user-friendly, and fast workflow combining existing tools for drug discovery through the integration of GWAS and QTL data. The Causeway tool is available at https://github.com/juliaapolonio/Causeway.

## 2 Methods

### 2.1 Overview of causeway

**Figure 1.**
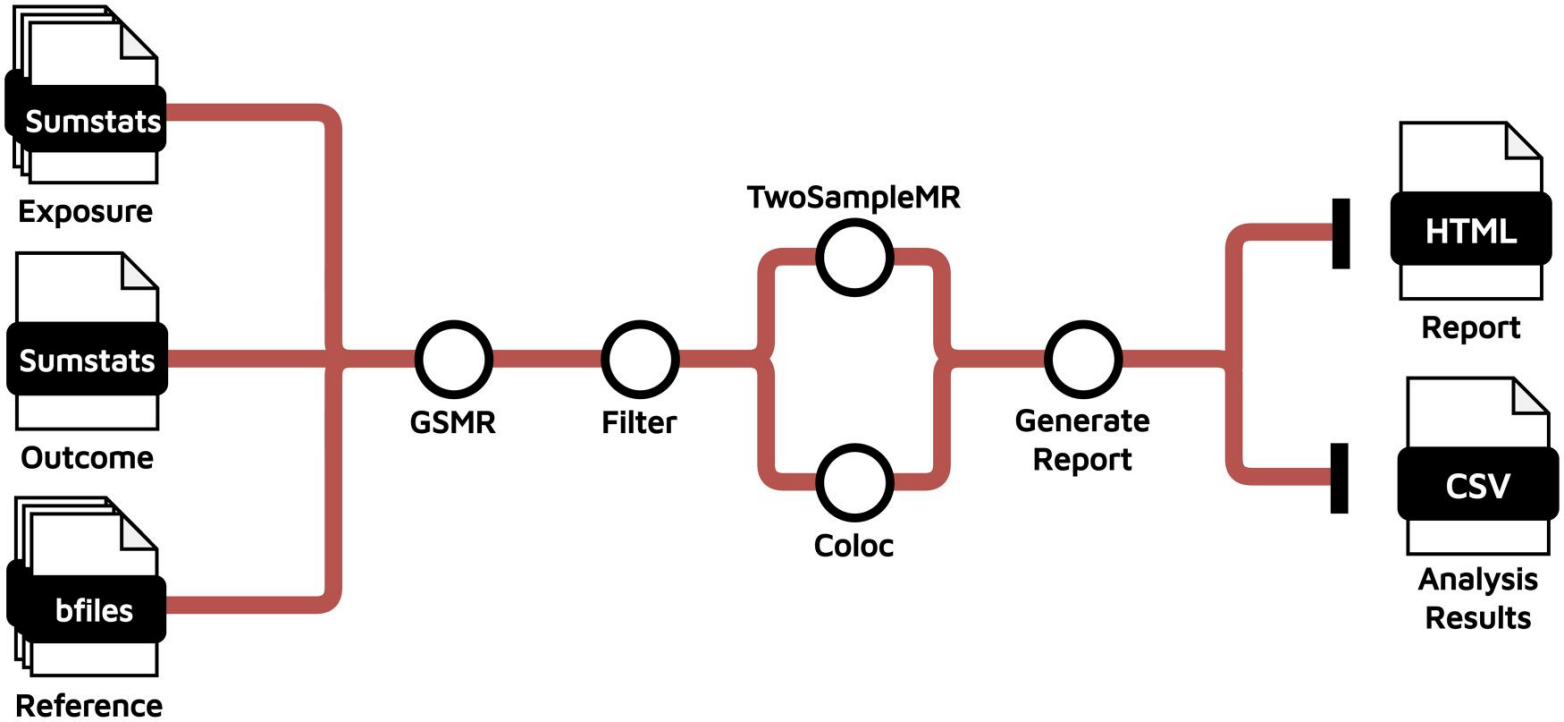
General overview of Causeway’s workflow. The pipeline requires as input summary statistics for exposure (split by gene for eQTLs) and outcome, as well as a reference panel with individual-level genotypes formatted in PLINK’s B-file. The pipeline runs GSMR/HEIDI-outlier per gene and calculates FDR-adjusted *P*-value correcting for multiple testing. An additional module filters genes retaining only those with significant GSMR results and reducing this way time and computational cost for subsequent steps: TwoSampleMR and colocalization analysis. The pipeline outputs a markdown with a summary of the analysis and regional/effect plots for each gene. A final set of modules parses those markdowns generating an HTML report with important findings and a CSV file with all results merged by exposure.

The pipeline comprises four analytical parts: (i) data preparation; (ii) MR; (iii) MR sensitivity analysis and co-localization; and (iv) report generation. It begins with running GCTA-GSMR (Zhu *et al.* 2018) using two local preprocessing modules and summary statistics for the outcome of interest (Fig. 1). These modules are a reference panel with individual-level genotypes (European 1000 Genomes) for estimating LD between IVs and molecular QTLs summary statistics (blood cis-eQTLs from eQTLGen dataset). Alternatively, the user can provide as input other sources of data. The HEIDI-outlier test included in GCTA-GSMR is also applied to filter out horizontal pleiotropic variants (PHEIDI < 0.01). Two additional local modules correct the GSMR *P*-values by applying the false discovery rate (FDR) method (FDR < 0.05) for multiple testing and select genes with significant causal effects on the phenotype of interest. In the second step, Causeway performs MR sensitivity analysis for GSMR significant genes using conventional MR methods implemented in the TwoSampleMR R package (Hemani *et al.* 2018) (Fig. 1). Those include inverse variance-weighted, weighted median, MR-Egger, and simple mode regression methods to ensure consistency in the directionality of effect sizes with GSMR betas; MR-Egger and inverse-variance weighted heterogeneity tests; MR-Egger intercept to assess horizontal pleiotropy; and Steiger directionality test. Furthermore, Causeway evaluates horizontal pleiotropy using the MR-PRESSO (Mendelian Randomization Pleiotropy RESidual Sum and Outlier) method (Verbanck *et al.* 2018). To account for multiple testing, *P*-values for each MR method are corrected using the FDR method. To assess whether the outcome (phenotype of interest) and exposure (e.g. gene expression) share a single causal variant within a given genomic region, Causeway also runs colocalization analysis using the R package Coloc (Wallace 2021) (Fig. 1). Under the assumption of a single shared causal variant within a genetic region, this function calculates posterior probabilities (PPs) for a set of five association hypotheses in a Bayesian framework: no association with either the exposure or the outcome, association with the exposure only, association with the outcome only, association with both traits but driven by different causal variants, and association with both traits driven by a shared causal variant. A posterior probability >0.8 for the shared causal variant hypothesis (PP > 0.8) is considered evidence for colocalization. In the third step, Causeway merges all results into a data frame (Fig. 1) and selects potential effector genes based on the following criteria: (i) significant FDR-adjusted *P*-values in GSMR analysis; (ii) significant FDR-adjusted *P*-values and consistent direction of effect across at least two additional MR regression methods; (iii) significant FDR-adjusted *P*-values for Steiger test of causal directionality; and (iv) no evidence of heterogeneity and pleiotropy.

Finally, Causeway generates forest (Supplementary Fig. S1), volcano (Supplementary Fig. S2), TwoSampleMR scatter (Supplementary Fig. S3), and regional (Supplementary Fig. S4) plots for visualization and stores results in an output folder, with results highlights in an HTML report. An additional important property of Causeway is easy reproducibility as the modules running environments are independent of the system used and users do not need to install any dependency apart from Nextflow. Furthermore, all dependencies are installed using version-locked containers stored in public repositories. The source code of the pipeline, as well as the documentation and a basic running example, are also available on GitHub.

### 2.2 Application

To evaluate Causeway’s performance, we used the European summary statistics of the latest meta-analysis of Type 2 Diabetes (T2D) (Suzuki *et al.* 2024) to find candidate causal genes for the disease using blood eQTLs from phase I cis-eQTLs of eQTLGen consortia (Võsa *et al.* 2021). The T2D summary statistics were filtered to retain only biallelic SNPs and formatted in GCTA-Cojo format using rsIDs as the identifier format for variants. Moreover, to assess whether the eQTLGen variants were strongly associated with the exposure, the F-statistics (beta^2^/se^2^) was calculated for each variant and weak IVs (F-statistic < 10) were filtered out. The reference data used was 1000 Genomes phase 3 European (GRCh37) genotype dataset in PLINK 2.0 p-files format (The 1000 Genomes Project Consortium 2015), which was converted to PLINK 1.9b-files format.

Using the Causeway pipeline, we identified 30 genes with potential causal effects on T2D (Supplementary Table S1 marked as TRUE in the candidate column), among which, 27 were within genomic regions previously associated with the disease and three were novel. More specifically, Causeway either provided additional evidence for causality of seven previously reported T2D genes or suggested alternative genes within 20 known T2D loci. Among those, three are included in the druggable genome (Finan *et al.* 2017): LPAR5 is a tier 2 druggable target, i.e. its product has known bioactive drug-like small-molecule binding, while AOAH and NUP210 are tier 3 druggable targets, i.e. their products are secreted or extracellular proteins, proteins with more distant similarity to approved drug targets and members of key druggable gene families not already including in tier 1 or 2. Among the novel loci for T2D, NDST1 is involved in the glycosaminoglycan metabolism pathway and glycosaminoglycans are known to suffer changes in various tissues during diabetes (Gowd *et al.* 2016).

The analysis was run using version 0.1.0dev of the software, in a cluster using a Slurm executor with singularity as the container platform. A total of 16 924 QTLs were used as exposure against one outcome, totaling 29 896 tasks composed of 16 924 from GSMR and 2024 from TwoSampleMR and Coloc, which concluded in 58 h of execution. Further details about task number, processing time and peak RAM consumption can be found in Supplementary Fig. S5 and S6 and Supplementary Table S2. This analysis can be replicated with the same data and parameters by following the instructions under https://juliaapolonio.github.io/Causeway/usage/#replication-of-the-paper-analysis.

## 3 Discussion

Causeway drastically reduces computational time by using a filtering step, which limits sensitivity analysis to the significant GSMR candidates—using the median execution times as an estimate, this resulted in an analysis 32% quicker than simply using TwosampleMR and Coloc outside of the pipeline (Supplementary Table S3). Moreover, the pipeline is user-friendly, well documented and can be executed in several platforms, requiring one command for installation and an additional one for the validation analysis. Furthermore, with a median peak RAM usage of 5.5 GB during validation, Causeway meets its goal of being accessible to low-resource computational environments. Finally, in addition to xQTL analyses, the pipeline is versatile and designed to enable the assessment of causal inference between any phenotypes using data in summary statistics format.

## Supplementary Material

vbaf110_Supplementary_Data

## Data Availability

The data underlying this article are available in diagram consortium webpage at https://www.diagram-consortium.org/downloads.html and can be accessed with Security Hash Algorithm (SHA1) number 6122209fff7c3f19a95d024c0ff75f0fe029d65f.
