# Supplementary material for: Causeway: a pipeline for genome-wide effector gene screening with Mendelian Randomization and colocalization
Source: Bioinform Adv. 2025 May 29;5(1):vbaf110. doi: 10.1093/bioadv/vbaf110 (PMC12161984; doi:10.1093/bioadv/vbaf110)
Supplement: vbaf110_Supplementary_Data [file vbaf110_supplementary_data.zip › Suppl_files_causeway.docx]

**Supplementary information**


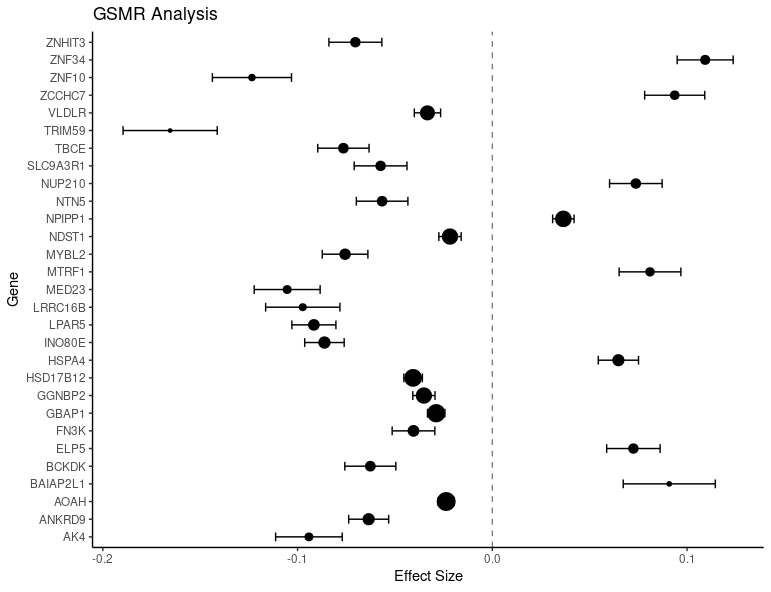


**Supplementary Figure 1 - Example of Causeway-generated forest plot showing the causal effects of prioritized genes on outcome of interest.** The circle size represents the variance of the effect size.


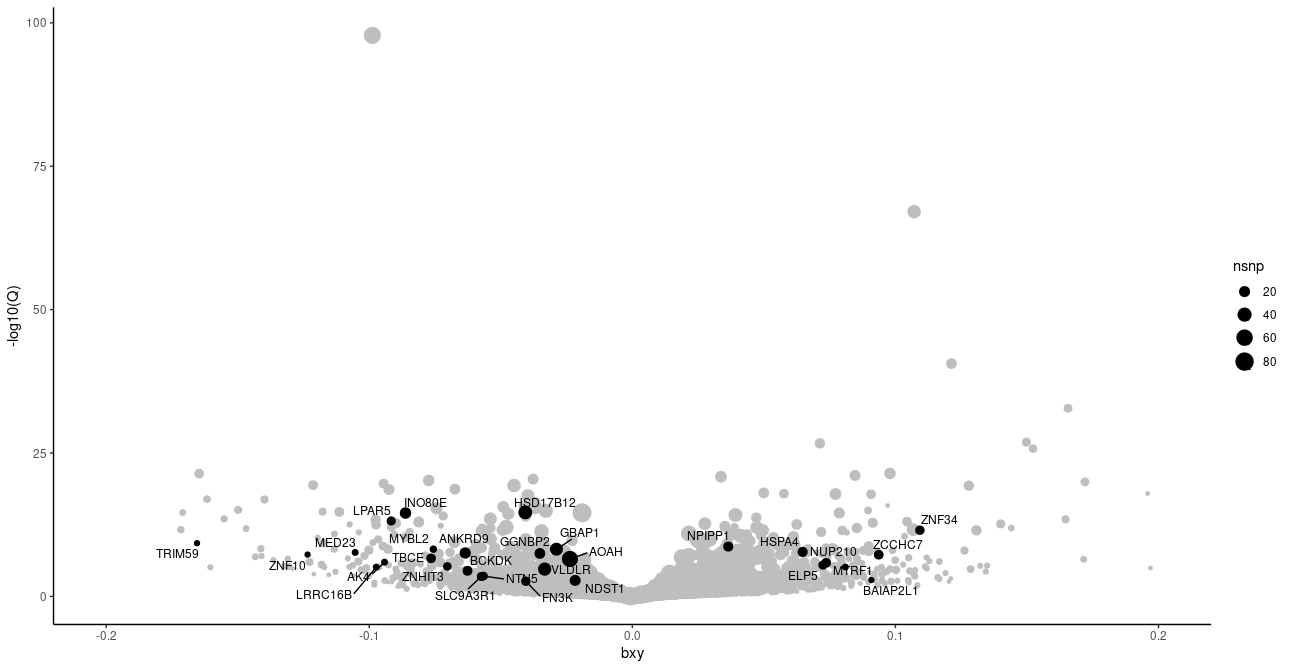


**Supplementary Figure 2** - **Example of Causeway-generated volcano plot with prioritized genes highlighted in black.** The x-axis represents the effect size of exposure on outcome and the Y-axis is showing the FDR-adjusted p-value. The circle size is proportional to the number of instrumental variables (IVs) used in GSMR analysis.


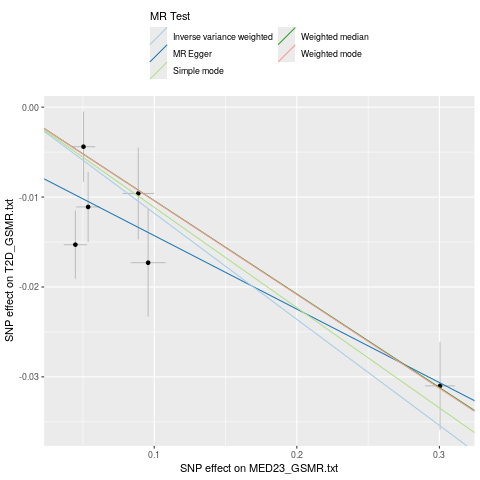


**Supplementary Figure 3 - Example of TwoSampleMR plot generated by Causeway showing the relationship of each IV effect on exposure against each IV effect on outcome across different MR regression methods.** The error bars correspond to 95% confidence intervals for the effect size.


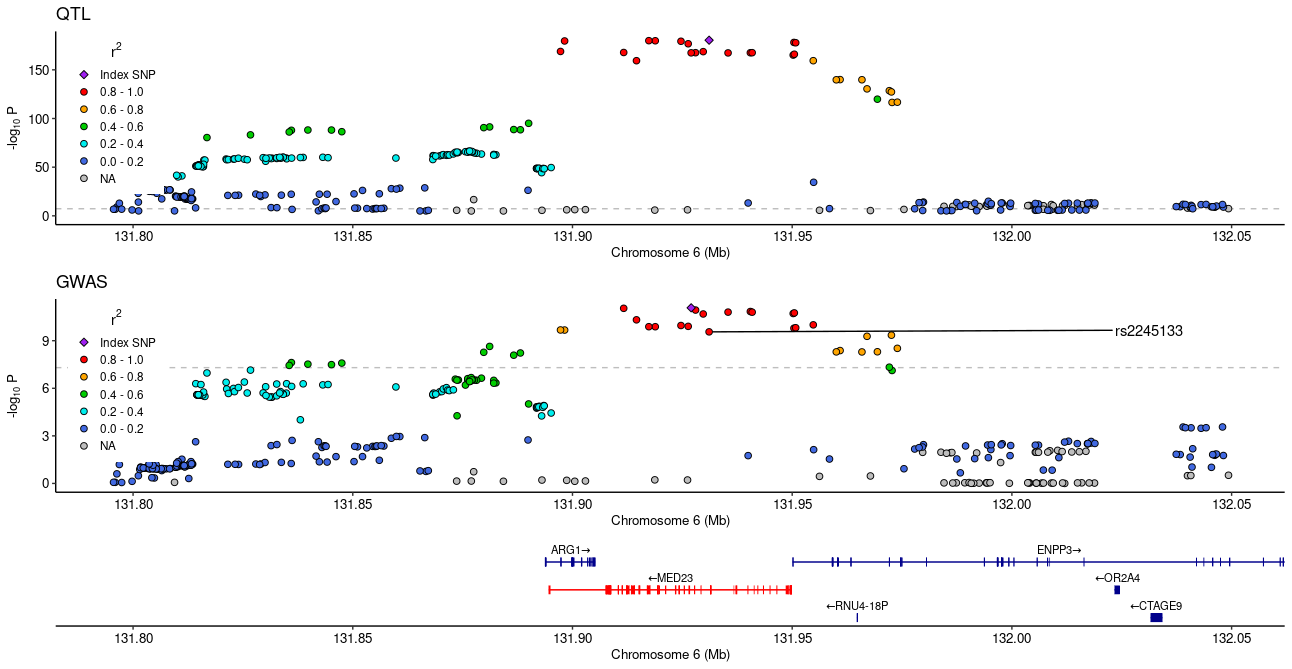


**Supplementary Figure 4 - Example of Bayesian colocalization as part of the Causeway pipeline.** This plot is generated using LocusZoom R package and shows regional plots of eQTL (upper) and outcome GWAS (lower) at a specific locus. The index variant in each plot is represented in purple and the colocalized variant is labeled with its rsID. The lowest part of the plot is a gene browser, with the target gene colored in red.


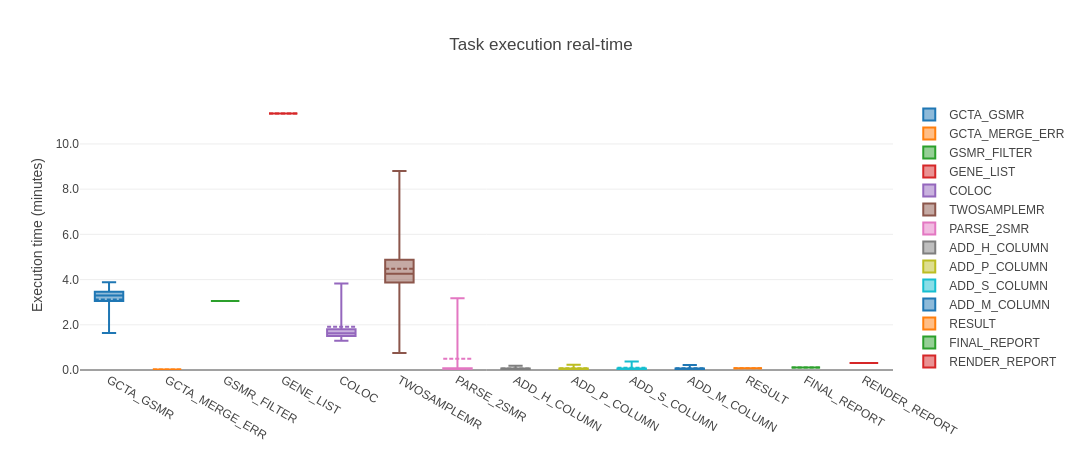


**Supplementary Figure 5 - Seqera Platform’s plot of task execution time by process.** Each process is represented by a different color. The most demanding processes are GENE_LIST, which transfers the GSMR significant genes to the workflow working directory and TWOSAMPLEMR, GCTA_GSMR and COLOC, which perform the analyses described in the paper.


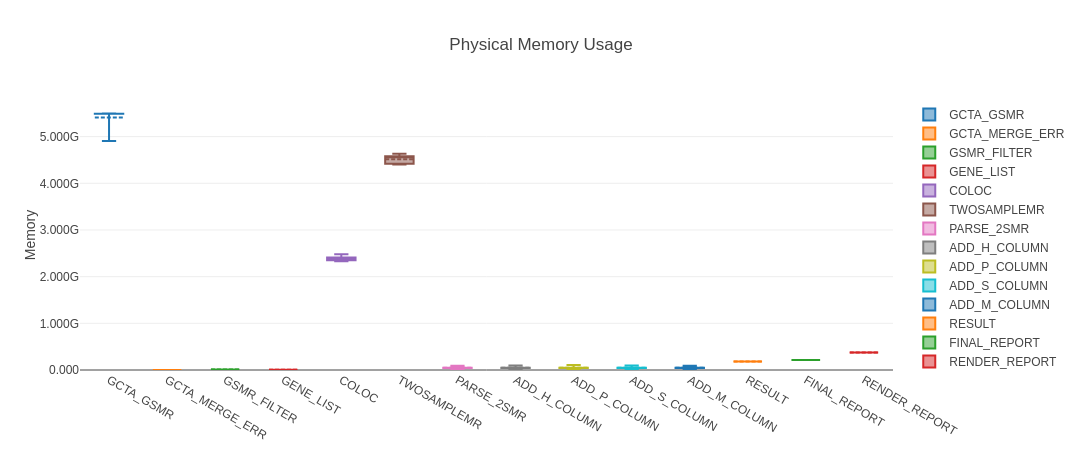


**Supplementary Figure 6 - Seqera Platform’s plot of RAM usage per task by process.** The most RAM-demanding tasks for the pipeline are GCTA_GSMR, TWOSAMPLEMR and COLOC. The highest individual peak of RAM doesn’t exceed 6 GB of RAM, meaning that this is the minimum requirement for a computer environment to run this pipeline.
